# Neglected extensive *Aspergillus* osteomyelitis of the pelvis, femur, and vertebra in an immunocompetent patient: A case report

**DOI:** 10.5339/qmj.2023.25

**Published:** 2023-12-12

**Authors:** Roop Singh, Anas Delair, Svareen Kaur, Monika Gupta

**Affiliations:** ^1^Department of Orthopaedic Surgery, Paraplegia & Rehabilitation, Pt. B.D. Sharma PGIMS, Rohtak, Haryana, India Email: drroopsingh@rediffmail.com ORCID iD: https://orcid.org/0000-0003-2800-0777; ^2^Dr. Baba Saheb Ambedkar Medical College, Rohini, New Delhi, India; ^3^Department of Pathology, Pt. B.D. Sharma PGIMS, Rohtak, Haryana, India

**Keywords:** Aspergillosis, osteomyelitis, femur, antifungal

## Abstract

*De novo Aspergillus* infections of the appendicular skeleton are rare. A 72-year-old female presented with pain and deformity in her left lower limb and an inability to bear weight that had persisted for the last six months. A femur biopsy confirmed the diagnosis of extensive *Aspergillus* osteomyelitis, and the patient was treated with amphotericin B and oral voriconazole. The patient died of COVID-19 after 2.5 months of treatment. A diagnosis of *Aspergillus* osteomyelitis may be delayed because of its varied clinical presentation. To the best of our knowledge, this is the first known case of de novo neglected and extensive (multisite, multibone) *Aspergillus* osteomyelitis in an immunocompetent patient. This case highlights the importance of awareness in patients and treating physicians of this rare infection and its early diagnosis to prevent extensive spread.

## Introduction

Fungal long-bone osteomyelitis typically affects individuals with impaired immune systems and is extremely rare in immunocompetent patients.^1^ *Aspergillus* osteomyelitis is a severe, incapacitating type of invasive aspergillosis.^2–4^ Direct trauma, previous surgery, hematogenous spread, or direct lung invasion can result in bone involvement.^3,4^ Patients with *Aspergillus* osteomyelitis may experience protracted pain, immobility, and loss of function.^3^ In clinical practice, diagnosing and managing osseous invasive aspergillosis poses great challenges.^3,5–7^ Diagnosis is confirmed by culture and/or histopathology. An early diagnosis significantly influences the outcome of *Aspergillus* osteomyelitis.^3–6^

Here, we describe a rare case of neglected extensive *Aspergillus* osteomyelitis of the pelvis, femur, and vertebra in an immunocompetent patient.

## Case

### Ethical statements

This study was exempt from review by the Institutional Review Board (IRB) as no IRB approval was required for case report publication in our institute. Written consent was obtained from the patient’s guardian to publish this case report.

A 72-year-old female presented to our orthopedic outpatient department with pain and deformity in her left lower limb and an inability to bear weight that had persisted for the last six months. Her medical history was insignificant; she had never received steroid/chemotherapy treatment. There was no history of a precipitating injury, surgical intervention, or substance abuse. The patient had no primary immunodeficiency, neutropenia, or chronic granulomatous disease history. She had intermittent fever but denied any gross loss of appetite or weight. She had received treatment for pain and fever from a local practitioner but never visited an expert care center. The treatment prescribed by the practitioner was in the form of two tablets (tramadol and calcium), which the patient had taken twice daily for the last two months. The patient had the prescription record.

Upon clinical examination, the patient had an average build. Physical examination revealed tenderness, mild swelling, and a deformity on the left proximal thigh. The overlying skin appeared smooth and shiny, without scarring or sinus discharge. Palpation revealed tenderness and crepitus of the proximal thigh.

**Figure 1.**
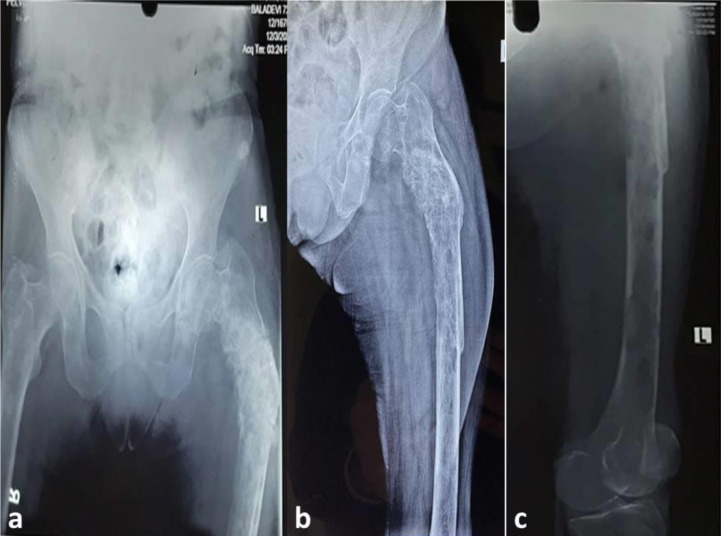
Plain radiographs of the pelvis (a) and left thigh (b, c) show multiple areas of osteolysis in the proximal femur with gross deformation and cortical breach. Numerous moth-eaten and permeative lucencies throughout the proximal and shaft of the femur can be appreciated. There is no apparent periosteal reaction or soft tissue component.

Radiographs of the pelvis, including both hips and left thigh, showed multiple areas of osteolysis in the proximal femur, with gross deformation and cortical breach. There were no noticeable periosteal reactions or soft tissue components (Figure 1). The chest radiograph was normal.

The patient was hospitalized, routine blood investigations were performed, and the results were within normal limits, except for a slightly elevated erythrocyte sedimentation rate of 38 mm/hour (range, 0–20 mm/hour) and a C-reactive protein level of 29 mg/L (range, <5.0 mg/L). Human immunodeficiency virus, hepatitis A, B, C, and E statuses were negative, and serum immunoglobulin and other blood tests were also normal.

**Figure 2.**
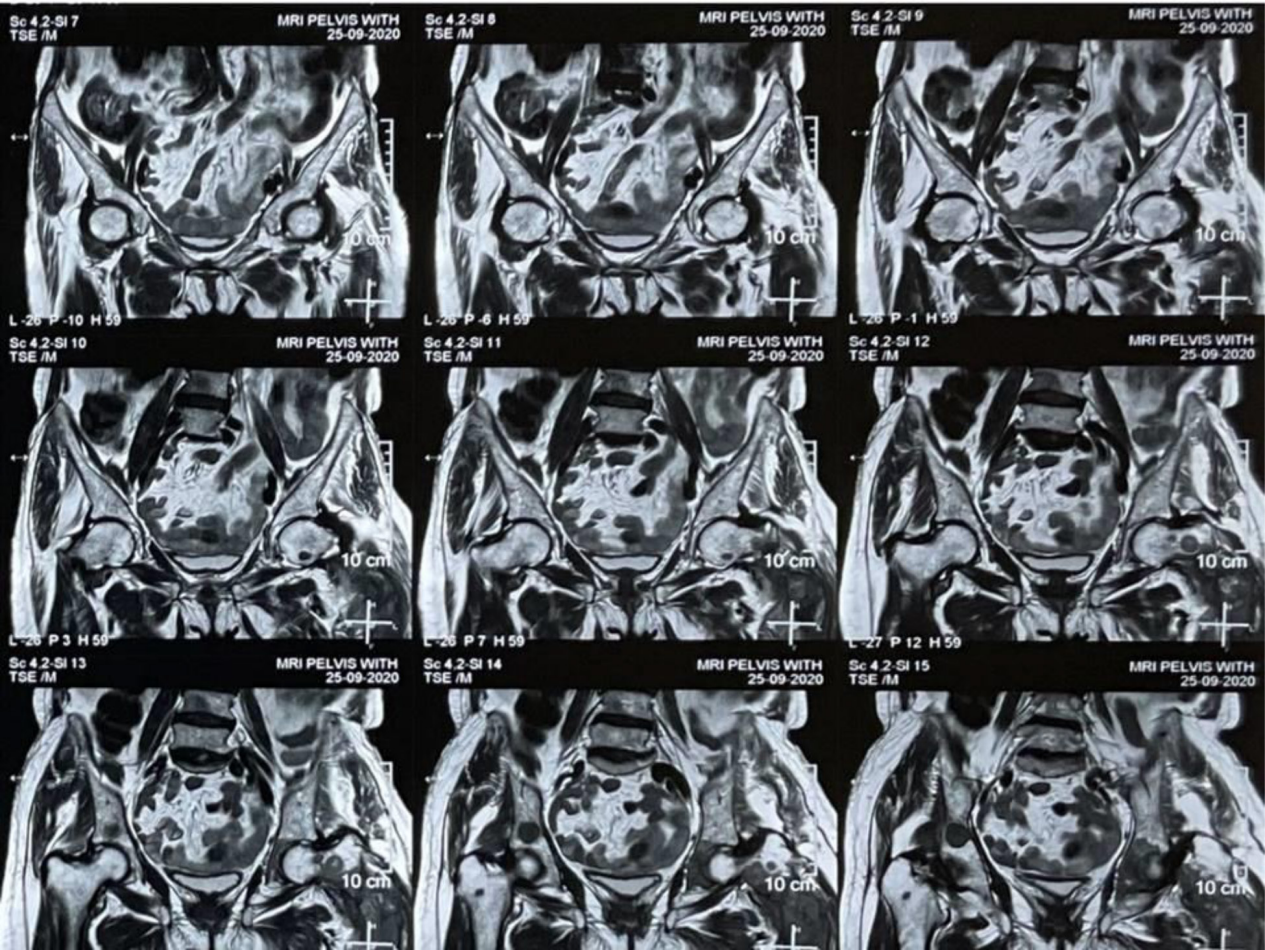
T2-weighted coronal images of the pelvic region show multiple rounded hypointense lesions scattered in bilateral pelvic bones, bilateral proximal femurs, and the lumbar fourth vertebra.

**Figure 3.**
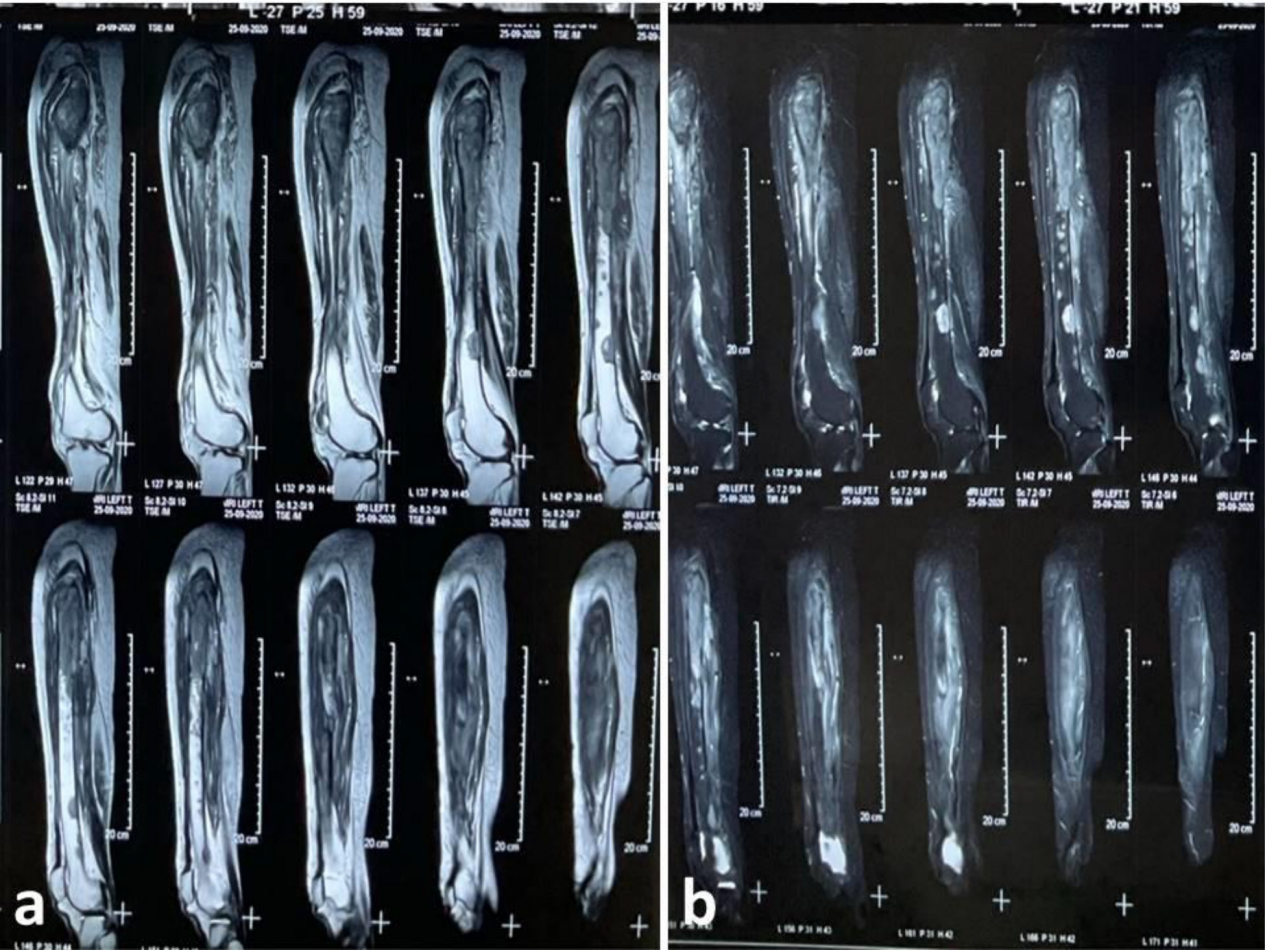
Magnetic resonance imaging sagittal images of the left thigh show a large heterogeneous T1-weighted (a) and fat-suppressed/ short tau inversion recovery (STIR) (b) hyperintense lesion involving the left proximal femur, neck, intertrochanteric region, and proximal shaft with areas of cortical break. Multiple other round lesions are present in the femur’s mid and distal shaft.

**Figure 4.**
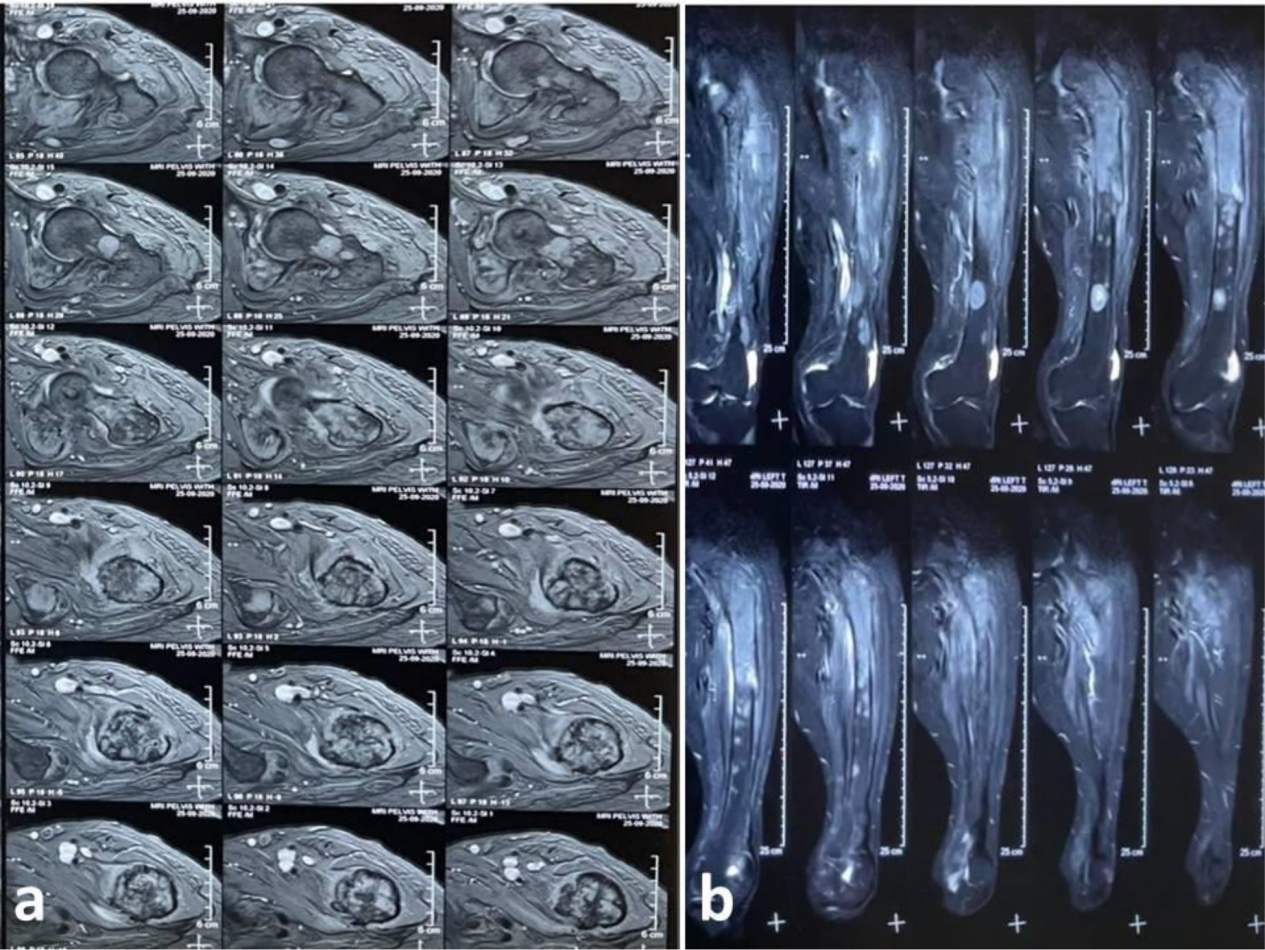
Magnetic resonance T1-weighted axial images of the proximal left femur show multiple hypodense lesions in the neck, intertrochanteric, and proximal femur (a), and T2-weighted coronal images of the left thigh showed perosseous edema and edema of the adjacent muscles. Posterior and medial cortexes are eroded, and soft tissue within the bone extends into the adjacent perosseous plane (b).

Magnetic resonance imaging (MRI) was performed, and it demonstrated multiple rounded T1- and T2-weighted hypointense and short tau inversion recovery (STIR) hyperintense lesions scattered in the lumbar fourth vertebra and mid and distal shaft of the left femur and bilaterally in the pelvic bones and proximal femurs. A large heterogeneous T1- and T2-weighted hypointense and STIR hyperintense lesion involving the left proximal femur, neck, intertrochanteric region, and proximal shaft, with areas of cortical break and periosseous soft tissue edema, were observed (Figures 2–4).

A core biopsy was then performed under local anesthesia. The bone specimens were sent for histopathological examination, Gram staining, acid-fast staining, and culture. Gram staining showed no bacteria and Ziehl–Neelsen staining was negative for acid-fast bacilli. Bacterial cultures for both pyogenic and acid-fast bacteria were negative. Still, gray-green velvety colonies with a narrow white border grew on potato dextrose agar, confirming the presence of *Aspergillus fumigatus*.

**Figure 5.**
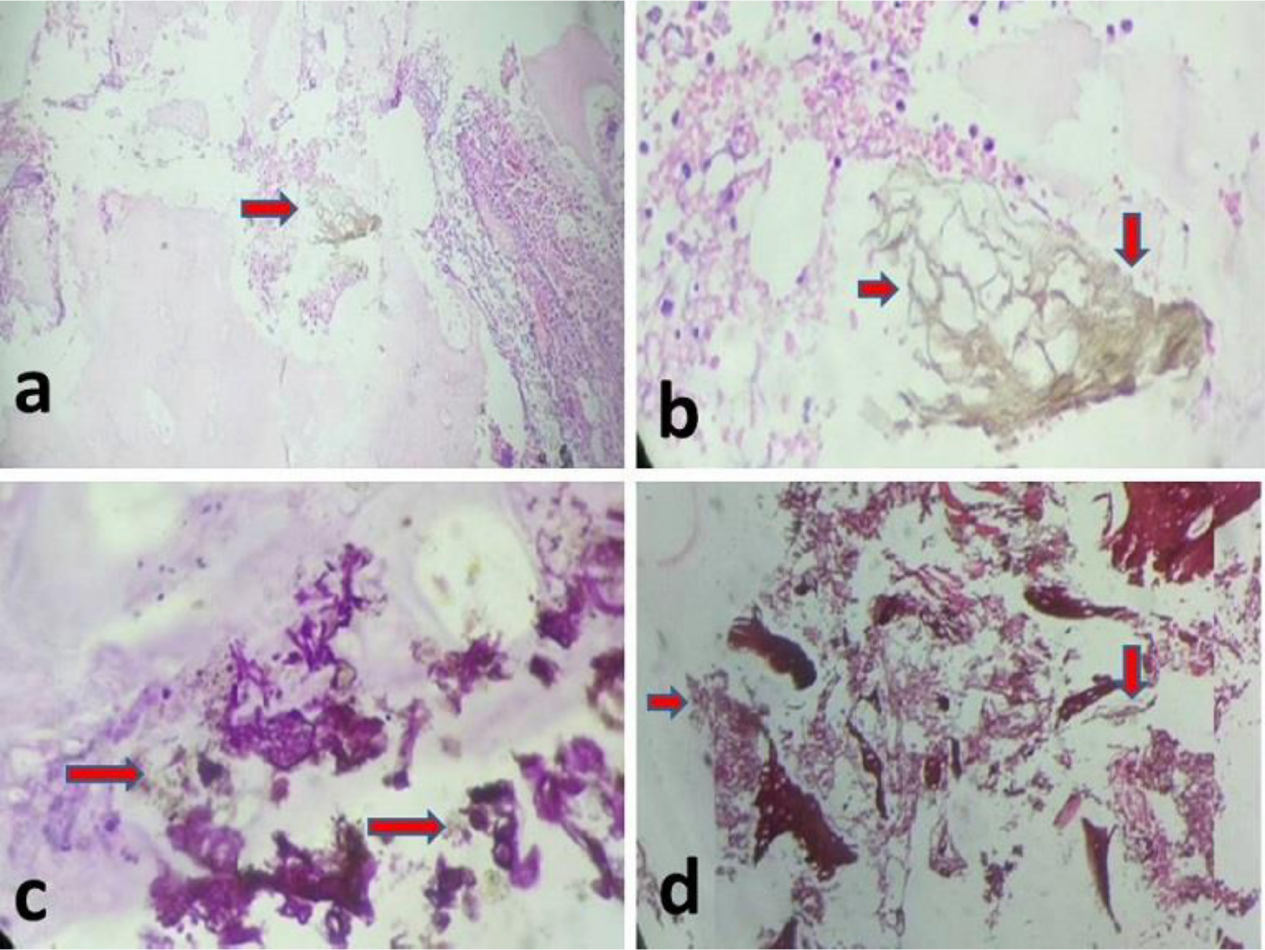
Hematoxylin and Eosin-stained section reveals necrotic bone, inflammatory granulation tissue, and narrow-branching septate hyphae (arrows) [100× (a) and 400× (b)]. (c) PAS (periodic acid–Schiff) stain reveals necrotic bone with branching septate fungal hyphae diagnostic of aspergillosis (arrow) (400×). (d) Gomori methenamine silver (GMS)-stained section reveals the characteristic grayish black branching septate hyphae of aspergillosis (arrow) (100×).

Histopathological specimens showed necrotic bone in hematoxylin and eosin-stained sections, inflammatory granulation tissue, and narrow-branching septate hyphae. After periodic acid-Schiff staining, branching septate fungal hyphae and necrotic bone were also observed. Gomori methenamine silver (GMS)-stained sections revealed the characteristic grayish-black branching septate hyphae of aspergillosis (Figure 5).

Based on the microbiological and histopathological evidence, a final diagnosis of *Aspergillus* osteomyelitis was made. Blood tests like the *Aspergillus* galactomannan test and beta-d-glucan test and advanced testing by genome sequencing to differentiate between various *Aspergillus* species still need to be done due to the non-availability of these modalities in our institute.

After discussions with an infectious disease consultant, antifungal treatment was initiated to first control the extensive spread, and surgical management was planned depending on the response to antifungal treatment. Intravenous amphotericin B (1 mg/kg/day) and oral voriconazole (200 mg every 12 hours) were initiated after baseline renal and liver function tests and electrocardiogram (ECG) were performed. The patient values and reference levels are shown in Table 1. The patient weighed 65 kg, and 65 mg of lyophilized amphotericin B (reconstituted with 10 mL sterile water supplied with the vials) was intravenously administered daily for two weeks in 500 mL of 5% dextrose solution over 3 hours after administering a test dose of 20 mL before the first dose. Therapeutic drug monitoring (TDM) for voriconazole was not performed because this capability was unavailable at our institute. The *Aspergillus* galactomannan antigen and β-D-glucan tests were also not performed because of non-availability. However, with the worsening of the coronavirus disease 2019 (COVID-19) pandemic, the patient was discharged upon request, receiving oral voriconazole (200 mg twice daily for three months) and skin traction. Regular telephone consultations and monthly outpatient department visits were also conducted. Routine complete blood count, ECG, and renal and liver function test results were within normal range after two months of follow-up. After 2.5 months of ongoing treatment, the patient died of COVID-19, and a post-mortem was not undertaken.

## Discussion

Bacteria are the most common causes of bone inflammation, whereas fungi are rare causes of osteomyelitis. *Candida* spp. and *Aspergillus* spp. are common causes of fungal osteomyelitis.^1^ Although *Aspergillus* osteomyelitis is rare, it presents a great challenge for treatment and has poor outcomes.^8,9^ The reported incidence of extensive osseous aspergillosis ranges from 1.8% (1990) to 5.6% (2005),^4,10^ with a mortality rate of up to 25%.^5,6^ immunosuppressed Patients are commonly affected by *Aspergillus* osteomyelitis. Spread from an adjacent pulmonary infection is observed mainly in children, whereas hematogenous spread is the usual route of long bone involvement in adults.^7,11–13^

Vertebrae are the most common sites (almost 50%) of *Aspergillus* infection.^3,5,6^ Cases of *Aspergillus* osteomyelitis in the appendicular skeleton are rare, with only a few case reports in the literature.^7^ Three recent reviews have been published on *Aspergillus* osteomyelitis.^3,5,6^ Gabrielli et al.,^5^ in a review of 310 cases from 1936 to 2013, reported an incidence of the site of involvement in the spine (49%), the base of the skull, paranasal sinuses, and jaw (18%); ribs (9%); long bones (9%); sternum (5%); and chest wall (4%). Gamaletsou et al.^3^ reported that the vertebrae (46%), cranium (23%), ribs (16%), and long bones (13%) were infected sites among 180 evaluable patients between 1947 and 2013. Koehler et al.^6^ analyzed 47 published cases of aspergillosis of bones and joints between August 2002 and 2013. The joints were infected in five, bones in 40, and combined bones/joints in two of the 47 patients. Koutserimpas et al.^11^ reported 68 bone infections in 63 patients, and five had two sites of infection. The authors reported that the ribs were most commonly affected (25 cases, 36.8%). None of these reviews or other published literature in English have ever reported such extensive *de novo Aspergillus* osteomyelitis of multiple bones as in the present case.

No clinical symptoms reliably distinguish between bacterial and *Aspergillus* osteomyelitis. *Aspergillus* species cause 11 Classic signs of osseous infection; however, the intensity depends on the host’s immune status, host response, and severity of infection. The diagnosis of fungal osteomyelitis in the early stages may be overlooked, particularly in immunocompetent individuals, unless cultures and histopathology confirm the diagnosis. The slow and protracted course of the disease results in late diagnosis in most cases. A delay of 32 weeks has been reported.^14^ The diagnostic conundrum is complicated by a clinical picture that is highly varied.^3^ As observed in the present case, patients with *Aspergillus* osteomyelitis may experience prolonged pain, immobility, and loss of function.

The literature has stated that in cases of *Aspergillus* osteomyelitis, identification of the causative microorganism is contingent upon a firm histology and culture diagnosis.^3,5,11^ A definitive diagnosis can be made by directly observing the branching septate hyphae on histopathological examination, using specific stains (methenamine-silver stain and periodic acid–Schiff stain), and recovering the organism in culture.^12,15,16^ In this case study, each of these investigations assisted us in diagnosing *Aspergillus* osteomyelitis.

*Aspergillus fumigatus* and *Aspergillus flavus* are the most pathogenic species that cause bone infections.^12,13^ Twenty more species, including *Aspergillus terreus, Aspergillus nidulans*, and *Aspergillus niger*, can also cause infection.^12,13,17^

Radiological investigations (MRI and computed tomography) add to the diagnosis, reveal the extent of involvement, and help plan the management modalities. MRI is a highly sensitive method for evaluating osteomyelitis.^18,19^ T1-weighted MRI images show edema, enhancement of the bone marrow, and replacement of the bright fatty marrow signal. T2-weighted images show marrow hyperintensity and enhancement and T2 hyperintense periosteal edema, which may be reactive to adjacent soft tissue infection.^18,19^ Marrow replacement on T1-weighted images is a good sign of marrow infection.^18^ These findings were also observed in the present case, and they further helped us determine the extensive involvement of the pelvic bones, proximal femurs, and lumbar vertebra, conclusions that could not be made using plain radiographs (Figures 1–4).

However, evidence-based guidelines for treating *Aspergillus* osteomyelitis still need to be developed. The treatment strategy is multimodal and consists of systemically active antifungal treatment, along with surgical debridement, decompression, and stabilization, depending on the extent and site of involvement.^3,5–7,11,20^ Surgical debridement includes thorough removal of all necrotic tissue (bone and cartilage), drainage of joints, and excision of sinus tracts.^3,5,7,11,20^ Unlike in chronic bacterial osteomyelitis, the role of surgery is still debatable in *Aspergillus* osteomyelitis.^20^ The present patient had extensive bone involvement; therefore, surgical debridement and reconstruction were postponed, and the disease was managed with antifungal treatment in the initial phase.

The present study has a few limitations, viz., the need for a large cohort or control group hampers the ability to compare outcomes and assess the effectiveness of specific treatments. The study could not describe detailed information on the treatment response, potential adverse effects of the administered antifungal agents, or long-term outcomes due to early death of the patient due to COVID-19. The strength of the present study is that it highlighted the importance of early diagnosis and management of aspergillosis to prevent extensive spread and pathological fractures. Future research with a more extensive patient population and comprehensive evaluation of treatment outcomes is warranted to enhance our understanding of this rare condition and optimize patient care.

The diagnosis of *Aspergillus* osteomyelitis may be delayed because of its varied clinical presentation. To the best of our knowledge, this is the first known case of de novo neglected and extensive (multisite, multibone) *Aspergillus* osteomyelitis in an immunocompetent patient. This case highlights the importance of awareness in patients and treating physicians of this rare entity and early diagnosis to prevent extensive spread.

## Conflict of Interest

No potential conflict of interest relevant to this article was reported.

## Funding

None.

## Figures and Tables

**Table 1.** Results of the blood investigations (renal and liver function tests).

	Observed value	Normal range
**Renal function test**		
Blood urea	33.34	13.0–43.0 mg/dL
Serum creatinine	1.2	0.7–1.4 mg/dL
Serum uric acid	4.93	4.40–7.60 mmol/L
Serum sodium	138.30	135–145 mmol/L
Serum potassium	4.04	3.5–5.5 mmol/L
Serum chloride	102.60	96–109 mmol/L
**Liver function test**		
Bilirubin total	0.42	<1.1 mg/dL
Bilirubin Direct	0.15	0–0.3 mg/dL
Bilirubin indirect	0.38	0.3–1.0 mg/dL
AST (aspartate transaminases)	15.8	<31.0 U/L
ALT (alanine transaminases)	13.8	<33.0 U/L
Alkaline phosphatase	48.8	40–120 U/L
Gamma-glutamyl transferases (GGT)	16.7	15–60 U/L
Total protein	7.4	6.0–8.0 g/dL
Albumin	4.40	3.5–5.5 g/dL
Globulin	3.06	2.5–3.5 g/dL
A/G ratio	1.44	1.5–2.5
